# ASO Author Reflections: Hematological Biomarkers of Survival in Cutaneous Melanoma

**DOI:** 10.1245/s10434-018-6913-8

**Published:** 2018-10-15

**Authors:** Alyss V. Robinson, Howard Peach, Ryckie G. Wade

**Affiliations:** 1 Faculty of Medicine and Health, Worsley Building, University of Leeds, Leeds, UK; 2 Department of Plastic and Reconstructive Surgery, Leeds General Infirmary, Leeds, UK

## Past

The incidence of cutaneous melanoma has risen faster than any other malignancy worldwide.^1^ Despite decades of research into tumor biology, as well as surgical and medical treatments, melanoma remains one of the most treatment-resistant cancers.

Biomarkers of disease help to stratify patients for different cancer treatments. The neutrophil–lymphocyte ratio (NLR) represents the host systemic inflammatory response to cancer and is strongly associated with survival, recurrence, metastatic burden, and treatment response.^2^ A recent systematic review of NLR in melanoma showed that a NLR > 2 was associated with a higher risk of death^3^; however, the majority of these data related to patients with metastatic disease, while the remainder had stage IIIc disease or very high-risk primaries. As the majority of patients (approximately 90%) have localized melanoma, the external validity of this biomarker research is weak, which provides the rationale for our multicenter work.

## Present

We observed that a low NLR (< 2.5) was associated with over twice the risk of death from melanoma.^4^ This finding contrasts with the observed direction of effect in most other solid organ cancers or advanced (stage III/IV) melanoma. This observation was compounded after stratifying patients by sentinel lymph node status, whereby patients with nodal metastases and a low NLR (‘poor immune responders’) had the worst survival. We suggest that because the NLR is a proxy for the host inflammatory response, a low NLR may represent a deficient immune response which might allow localized disease to propagate. This finding is potentially clinically important because if we could identify these ‘poor immune responders’ at baseline, adjuvant immunotherapy could be better utilized.

Although we made substantial efforts to mitigate the risks of methodological biases in our work, further prospective work is needed to define the associations between localized melanoma and hematological biomarkers.

## Future

The use of adjuvant immunotherapy for resected stage III/IV disease is now commonplace. Trials of neoadjuvant immunotherapy in high-risk resectable and oligometastatic melanoma prior to resection are now underway,^5^ which is both interesting and concerning because immunotherapy induces serious adverse events in approximately 15% of patients receiving ipilimumab monotherapy, 2% receiving pembrolizumab, and approximately 5% receiving nivolumab. Therefore, we suggest that the selection of patients for neoadjuvant immunotherapy is of paramount importance to prevent treatment-related harm. At the time of diagnosis, we have shown that hematological biomarkers might help to identify ‘poor immune responders’ who stand to gain the most from neoadjuvant immunotherapy.

Ultimately, multivariable modeling of biomarkers (hematological, histopathological, genomic, imaging, and phenotypic, etc.) will improve the selection of patients for different treatments. To implement this type of ‘personalized cancer treatment’ and improve the management of melanoma, we must share data derived from international collaborations and exploit modern computational methods of modeling big data.
